# Patients’ perspectives can be integrated in health technology assessments: an exploratory analysis of CADTH Common Drug Review

**DOI:** 10.1186/s40900-016-0036-9

**Published:** 2016-06-07

**Authors:** Sarah Berglas, Lauren Jutai, Gail MacKean, Laura Weeks

**Affiliations:** 1 CADTH, Ottawa, Canada; 2 London School of Economics, London, UK; 3 Department of Community Health Sciences, University of Calgary, Calgary, Canada

**Keywords:** Patient engagement, Health technology assessment, Evaluation, CADTH, Common Drug Review

## Abstract

**Plain language summary:**

In Canada, the CADTH Common Drug Review helps ensure that scarce health care resources are used to fund the most effective drugs. Clinicians, researchers, payers, and patients all have important, but potentially different, ideas on what should be considered, to determine a drug’s value. Since 2010, CADTH has invited patient groups to contribute their perspectives to the Common Drug Review. We explored whether, and how, insights offered by patient groups are integrated into assessment reports and Recommendations by the CADTH Canadian Drug Expert Committee.

After examining 30 completed drug assessments, we found that:

- Patient insights are used by CADTH reviewers to frame an assessment and are used by the expert committee to interpret the evidence.
- Drug trials do not always measure outcomes that patients consider important.
- Survival, symptom relief, the process of recovery, and maintaining health are all important aspects to consider when determining value during health technology assessments.

**Abstract:**

**Background**

Since 2010, Canadian patient groups have contributed to the CADTH Common Drug Review (CDR). CADTH conducts health technology assessments of new drugs to support publicly funded drug plans’ reimbursement decisions. We explored whether, and how, patient insights were integrated into assessment reports and Recommendations by the CADTH Canadian Drug Expert Committee (CDEC).

**Methods**

We descriptively analyzed 30 consecutive assessments. One researcher identified a set of issues, insights, and desired treatment outcomes provided by patient groups for each included drug assessment. We tracked the presence of each identified patient insight in the relevant assessment protocol, in clinical trials as reported in the assessment, and in the CDEC Recommendations. Additionally, patient insights were categorized by topic and grouped into a three-tier framework to explore the observed juxtaposition between immediate treatment outcomes as seen in clinical trials and the insights from patients living with a chronic condition.

**Results**

In 30 drug assessments, 119 patient insights were identified. Of these insights, 89 were included in assessment protocols; 61 in reported clinical trial data; and 67 insights were reflected upon within the CDEC Recommendations. Patient insights within the first framework tier (health status achieved) were frequently included in all aspects of CDR assessments. Within the second tier (progress of recovery), although two-thirds of patient insights were included in protocols, only one-third was reflected in reported trial data or in CDEC Recommendations. Insights within the third tier, which address the long-term consequences of illness and treatment, were even less frequently addressed in all aspects of CDR assessments.

**Conclusions**

Patients’ perspectives need not be “considered” in isolation. Patient insights are used by CADTH reviewers to frame an assessment and used by CDEC to interpret the evidence. As health technology assessments should address the indirect and unintended consequences of a technology, as well as its direct and intended effects, drug assessments should consider the progress of recovery and sustainability of health, in addition to survival and immediate health achieved.

**Electronic supplementary material:**

The online version of this article (doi:10.1186/s40900-016-0036-9) contains supplementary material, which is available to authorized users.

## Background

In Canada, the responsibility for providing health care falls to each province and territory. As a result, there are multiple publicly funded drug plans that cover the cost of some medicines, or provide them at a subsidized cost to those without private drug coverage. The pockets of taxpayers are not bottomless, so plan managers are faced with difficult decisions about which drugs to reimburse, for whom, and for how long. Since 2003, the CADTH Common Drug Review (CDR) has assisted the participating plans in making these decisions by providing an assessment of new drugs’ clinical and cost-effectiveness, as compared to currently available treatments.

In 2010, CADTH created opportunities for patient groups to contribute to health technology assessments (HTAs) in the CDR process. Why? Patients have unique knowledge about the disease and treatment that can and should inform an HTA [1]. They are able to identify potential benefits and harms that are important to them and knowledgeably comment upon whether the benefits outweigh the harms that the treatment may have on other aspects of their lives [2]. Studies from the United Kingdom indicate that people with a chronic illness spend around 10 h per year with health professionals, whereas they spend 6,000 h self-managing their condition [3]. Illness, especially chronic illness, is one part of that person’s life and their families’ lives. In the “lifeworld,” the illness, as lived, may differ from the disease as described in the evidence-based guidelines and the outcomes measured in clinical trials. [4]

Another reason for involving patients in HTA is to increase transparency and openness in public decision-making [5]. As it is impossible to please everyone when making difficult reimbursement decisions, building trust and respect in the decision-making process is essential. A fair process for resource allocation requires transparency about the reasons that play a part in decisions [6]. The cry “No decision about me, without me” conveys a basic democratic principle: to involve those affected by the decision in the decision-making process [7]. Canadian patient groups clearly have an interest, and have sought involvement, in HTA [8].

Between 2010 and 2015, 114 patient groups provided 297 patient input submissions to CDR, contributing to 142 reimbursement recommendations [9]. For each new drug assessment, CADTH invites patient groups to share their perspectives via a written template [10]. Patient groups respond to questions to provide their perspectives regarding the impact of the disease on patients and their families, experiences with current therapies, and hopes regarding and/or experiences with the drug under assessment. Patient group input is sought early in the process, so patient insights can be included within the assessment protocol and the assessment reports. These reports go to the CADTH Canadian Drug Expert Committee (CDEC) for independent review and are used as the basis for deliberation when making a reimbursement recommendation. The 14-member committee is composed of economists, clinical specialists, pharmacists, epidemiologists, and two public members. The two public members of CDEC provide a lay perspective and have the additional responsibility of presenting patient perspectives, using the written submissions from patient groups, at the start of CDEC’s deliberations. [11]

CDR assessment reports and CDEC documentation include stand-alone sections describing insights and issues raised by patient groups in their written submissions. These sections demonstrate that CADTH reviewers and CDEC members have heard patient voices. However, it is more difficult to identify specifically how CADTH reviewers and CDEC members then respond to patient insights. While CDEC members are asked to consider patient insights, along with insights offered by clinical experts, the recommendation framework used by CDEC does not refer explicitly to patient values or preferences [11].

Despite rapidly expanding approaches to patient involvement, to date there has been little examination of the impact of patient involvement on HTA [12, 13]. Patient groups and their donors, HTA agencies and their funders, and academic groups understandably want to know how patient groups’ contributions have been used and whether and to what extent they influence the assessment process and decision-making [14]. Patient groups may have hoped, for example, that more drugs would be recommended for reimbursement with the introduction of patient input to CDR [15]. However, two external analyses on the impact of patient input to CDR did not show a change in the proportion of drugs recommended for reimbursement [15, 16]. This is perhaps not surprising, given that each CDR recommendation is based upon an appraisal of overall clinical effectiveness involving multiple clinical outcomes; insight provided by clinical specialists; cost-effectiveness, which varies depending upon the comparators used, price submitted, and assumptions made in the economic model; and patients’ perspectives on current treatment needs and expected improvements. That is, patient insights are one of many considerations that can shape a reimbursement recommendation.

Informal analysis over time has revealed explicit references to information provided by patient groups within different sections of CADTH assessment reports and in different sections of CDEC’s Recommendations. Anecdotally, these explicit references have increased in frequency and length over time. However, improved understanding of how patient insights and issues are used in each assessment requires careful study of key CDR documents to trace implicit references to the insights that patient groups contributed. The purpose of this descriptive study was to explore whether, and how, insights offered by patient groups are integrated into CDR reports and CDEC Recommendations.

## Methods

We descriptively analyzed a consecutive sample of reports associated with 30 CDR drug assessments completed between December 2012 and June 2014. To develop the sample, we began with the most recently completed drug assessment (June 2014) and worked consecutively backward, until no new insights or issues could be identified (i.e., saturation). Drug assessments were excluded if no patient input was received (nine assessments). (See Table 1: Included CDR Assessments.)

**Table 1:** Included CDR Assessments

Drug	Indication
Simeprevir (Galexos)	Hepatitis C, chronic
Onabotulinumtoxin A (Botox)	Migraine, chronic
Rotigotine (Neupro)	Parkinson disease
Eplerenone (Inspra)	Reduce risk of heart failure
Aclidinium bromide (Tudorza Genuair)	Chronic obstructive pulmonary disease
Tocilizumab (Actemra)	Polyarticular juvenile idiopathic arthritis
Golimumab (Simponi)	Ulcerative colitis
Ingenol mebutate (Picato)	Keratosis, actinic
Somatropin (Genotropin)	Growth hormone deficiency, adult
Ocriplasmin (Jetrea)	Vitreomacular adhesion
Lurasidone (Latuda)	Schizophrenia
Ulipristal acetate (Fibristal)	Uterine fibroids
Azilsartan medoxomil (Edarbi)	Hypertension
Azilsartan medoxomil + chlorthalidone (Edarbyclor)	Hypertension
Perampanel (Fycompa)	Epilepsy, partial-onset seizures
Everolimus (Afinitor)	Angiomyolipoma associated with tuberous sclerosis complex
Zolpidem tartrate (Sublinox)	Insomnia, short-term treatment
Dimethyl fumarate (Tecfidera)	Multiple sclerosis, relapsing
Nebivolol (Bystolic)	Hypertension
Adalimumab (Humira)	Juvenile idiopathic arthritis
Abatacept (Orencia)	Rheumatoid arthritis
Eculizumab (Soliris)	Atypical hemolytic uremic syndrome
Palonosetron hydrochloride (Aloxi)	Chemotherapy induced nausea
Glycopyrronium (Seebri)	Chronic obstructive pulmonary disease
Elvitegravir + cobicistat + emtricitabine + tenofovir disoproxil fumarate (Stribild)	HIV-1 infection
Pirfenidone (Esbriet)	Pulmonary fibrosis
Collagenase clostridium histolyticum (Xiaflex)	Dupuytren’s contracture
Ivacaftor (Kalydeco)	Cystic fibrosis (G551D mutation)
Apixaban (Eliquis)	Thromboembolic event prevention (atrial fibrillation)
Fidaxomicin (Dificid)	*Clostridium difficile* infection

One researcher (LJ) identified a set of issues, insights, desired treatment outcomes, and other ideas raised by patient groups for each included drug assessment, which were then tracked through the CDR process. For simplicity, we will refer to these as “patient insights.” To develop the set for each drug assessment, LJ coded relevant insights presented within the Summary of Patient Input and labelled each with a short descriptive phrase; for example, “prevention of blindness,” “more tolerable side effects,” and “treatment that extends beyond symptomatic and emergency relief.” Anywhere from zero to eight patient insights were identified for each assessment, with four or five insights per assessment being the most common. To confirm whether the appropriate insights had been identified, LJ consulted existing CDEC discussant reports, which include a section on “Key Issues Identified by Patient Groups.” These reports are prepared by the CDEC public members, based on their reading of the original patient input submissions. Additional insights were added if they had not been previously identified.

Next, for each of the 30 included assessments, LJ tracked the absence or presence of each identified patient insight in the three CDR documents — assessment protocols, assessment reports, and CDEC Recommendations — to determine whether the patient insights were included. Only insights identified by patient groups as important for that assessment were tracked. This analysis is not intended to be a critique of any individual drug assessment. Instead, our analysis seeks to understand how patients’ perspectives are integrated into CDR, in general. Table 2 shows a summary of key documents in the CDR process.

**Table 2:** Key documents in the CDR process

Summary of Patient Input	For each assessment, two CADTH reviewers consolidate and summarize information submitted via the patient input templates from all contributing patient groups. Contributing patient groups are asked to validate the summaries produced by CADTH to ensure all insights have been accurately captured. These summaries are included in the assessment reports.
CDR Assessment Protocol	The protocol is developed by a CADTH review team that includes three CADTH clinical reviewers, two health economists, a methodologist, and two external clinical specialists. The protocol follows the population, intervention, comparison, outcome (PICO) format.
CDR Assessment Report	CADTH reviewers analyze, appraise, and summarize all relevant trial data submitted to CADTH for the review. The trial design and outcome selection decisions rest with the pharmaceutical company and other external trial sponsors. A review of the assessment report allowed us to determine the extent to which patient insights were addressed in clinical trials performed by others.
CDEC Recommendation	This publicly available document describes the recommendation resulting from CDEC deliberations and the reasons for that recommendation. While it does not capture the full discussion, it provides a record of the considerations CDEC has agreed to highlight. CDEC Recommendations were analyzed to ascertain the extent to which patient insights were incorporated into the CDEC deliberation.

*CDEC* CADTH Canadian Drug Expert Committee, *CDR* CADTH Common Drug Review

To facilitate presentation of the results, patient insights identified within the included assessments were categorized by topic, and these 14 categories were then further grouped into a three-level framework for a better understanding of the potential value of a new medicine from a patient perspective. This framework, Michael Porter’s Outcome Measures Hierarchy, recognizes that patient experience extends beyond a medical intervention, to the process of recovery and maintenance of the achieved health state [17]. Using the framework allowed us to quantify the juxtaposition we observed between immediate outcomes of treatment seen in clinical trials and the experiences of, and concerns raised by, patients living with a chronic condition.

Patient insights were grouped within the first tier if they related directly to the ability of the treatment to prevent death or relieve disease symptoms. This might include preventing seizures or lung exacerbations, or improving fatigue for those with chronic illnesses, so that patients retain their current level of health. Insights related to receiving treatment, or adapting to life with treatment for a chronic condition, were grouped within the second tier. Over time, the burden of side effects, waning of effectiveness, willingness to receive treatment, and ability to pay for treatment contribute to a patients’ ability to recover or maintain health. (In Canada, the cost of drugs and other medical treatment received in hospital or acute-care setting are not borne by patients. In contrast, the cost to a patient’s family for drugs received outside of hospital will vary depending upon the family’s drug coverage plan.) Insights relating to long-term consequences of treatment or psychosocial consequences of being ill, such as willingness to trust your body or ability to live independently, were grouped in the third tier on sustainability of health, which encompasses recurrences of the original disease or new health problems created as a consequence of treatment. We analyzed our data at both the drug assessment level and for each category of patient insights.

As this study did not involve human participants or analysis of confidential personal data, we did not seek approval from a research ethics board. Preliminary findings were shared with patient group members of the CADTH Patient Community Liaison Forum [18]. Their questions and advice helped shaped further analysis and presentation of results.

## Results

A total of 119 patient insights were identified from patient input summaries from 30 drug assessments, which were grouped into 14 categories within the three tiers of Porter’s Outcome Measures Hierarchy framework [17]. Scan down Table 3 to see different categories of patient insights, within the tiered framework. Read across Table 3 to see how many insights for that category that were identified within the Patient Input Summary were included within CDR assessment protocols; included in clinical trials, as reported in CDR assessments; and included in CDEC Recommendations.

**Table 3:** Inclusion of patient insights into CDR process

Category	Description	Patient insights included in:			
		Patient summary	CDR protocol	Drug trials	CDEC Recs.
Tier 1: Health status achieved or retained					
Symptom relief	Improvement of specific symptoms, such as fatigue; seizure frequency; attack severity; ability to breathe, to eat, sleep, or move.	25	21	22	17
Health-related quality of life	General improvement in quality of life, as measured using standard general or disease-specific quality-of-life scales.	18	18	11	13
Target root cause	Treatment that targets the root cause of the condition, provides a cure, or extends beyond symptom control.	7	7	6	6
Long life	Increased survival, including slower disease progression, sustained remission, and fewer disease-related deaths.	5	5	3	5
Tier 1 subtotal		55	51	42	41
Tier 2: Progress of recovery					
Fewer side effects of treatment	Side effects of the treatment anticipated to be fewer, or less severe than current medications.	16	13	5	7
Ease of adherence	Ability and willingness to continue taking a medication. Can be influenced by number of pills, ability to self-inject, or contraindications while on the medication.	11	7	2	4
Alternative treatment	Alternatives needed if there is wide variability in individual response to treatment or if treatment efficacy wanes over time.	4	3	3	4
Fewer treatment supports	Reduction in the need or use of rescue medication or supplementary therapies to relieve symptoms.	3	2	1	0
Treatment duration	Shorter duration of therapy and/or recovery than current therapies.	3	1	1	1
Avoid hospitalization	Avoidance of surgery and other procedures requiring hospitalization of the patient.	6	6	3	3
Cost	Reduction in the cost borne by individual patients in accessing treatment.	6	0	0	3
Tier 2 subtotal		49	32	15	22
Tier 3: Sustainability of health					
Psychosocial quality of life	Mental, physical, and emotional ability to engage in daily life activities such as work, meeting friends, and caring for children.	7	4	2	1
Avoid further disease	Prevent disease transmission or subsequent illness, such as infection, infertility, blindness, or related cancers.	6	2	2	3
Independence	No longer dependent upon a caregiver to receive treatment or for basic self-care.	2	0	0	0
Tier 3 Subtotal		15	6	4	4
Totals		119	89	61	67

*CDEC* CADTH Canadian Drug Expert Committee, *CDR* CADTH Common Drug Review, *Rec.* Recommendation document

### Assessment protocol

Most of the 119 patient insights (89; 75 %) were included in CDR assessment protocols. Insights that fell within Porter’s first tier (health status achieved or retained) were most frequently included in protocols (51/55; 93 %), and insights that fell within the third tier (sustainability of health) were least frequently included (6/15; 40 %). Because the patient input template asks for comments on disease symptoms, health-related quality of life, treatment side effects, and adherence, it is unsurprising that insights within these categories were most frequently shared by patients and incorporated in protocols. Of the 14 categories of patient insights (see Table 3), only two were not included in the assessment protocols. Cost to the patient was likely not included, as it is outside of the scope of the assessment. Independence was the only other category not included. We do not know why.

All drug assessments for which there were patient insights included at least one or more insights in the relevant protocols; 23 of 30 (77 %) assessments included at least one-half of the insights offered by patients. All patient insights were included within eight (27 %) of the 30 assessment protocols. See Additional file 1, Table S1, for an overview at an assessment level.

### Clinical trials, as reported within CDR assessments

Upon studying the CDR assessment reports, we found there were drug trial data to address half (61/119; 51 %) of the patient insights. Patient insights within Porter’s first tier were most commonly studied in clinical trials (42/55; 76 %). Data on specific symptom relief (22/25, 88 %) and a mechanism of action to target the root cause of the disease (6/7; 86 %) most closely aligned to patient identified needs. In contrast, insights within other tiers were less often studied, with 15 of 49 (31 %) tier-two insights and four of 15 (27 %) tier-three insights included in clinical trials. Side effects of current treatments (for example, dependency, drowsiness, cognition) (5/16; 38 %) and the ability of patients to adhere to therapy (2/11; 18 %) were not frequently assessed in trials of new drugs.

Again, in all drug assessments for which patient groups provided insights, at least one or more patient insight was reported in the drug trials; 14/30 (45 %) assessments included at least one-half of all patient insights. One of the 30 assessments (3 %) had trial data to address all patient insights related to that assessment.

### CDEC Recommendations

Our analysis found that in their Recommendations documents, the CDEC members highlighted slightly more than half (67/119, 56 %) of patient insights. The bulk of the insights (41/55; 75 %) considered by CDEC fall within Porter’s first tier: health status achieved. Less than half of the insights (22/49; 45 %) identified by patient groups that fell within the second tier, and approximately one-quarter of insights within the third tier (4/15; 27 %), were discussed at CDEC. In addition, CDEC considered all insights related to how the new treatment provided a needed alternative (4/4; 100 %), due to waning effectiveness or wide variability of individual response to treatment, and all concerns (5/5; 100 %) related to mortality. The cost borne by individual patients in accessing treatment was considered in half (3/6; 50 %) of the assessments in which it was offered as an insight.

The majority (50/67; 76 %) of patient insights commented upon by CDEC members had trial data to inform that discussion. Without data, discussion is limited, although lack of evidence can be highlighted as a research gap within the published CDEC Recommendations. Nine patient insights without adequate trial data were highlighted as research gaps. These patient insights related to symptom relief, treatment side effects, discontinuing treatment supports, avoiding hospitalization, avoiding further disease, and mortality.

In one assessment, no patient insights were identified within the Recommendations documents, although two were included in the protocol. In all other assessments for which there were patient insights, at least one or more insight was recorded in the Recommendations documents; 17/30 (57 %) assessments identified at least one-half of patient insights. All insights were identified in three of 30 (10 %) assessments.

In four assessments, a patient insight was noted in the CDEC Recommendations, although that insight was not included in the relevant assessment protocol, and was without trial data. The insights related to the need for an alternative therapy for treatment-resistant patients; reducing vulnerability to recurring infections; the impact of an oral formulation; and the need for a simple treatment regimen. These insights may have come from a committee member’s own reading of the Patient Input Summary or original patient input submission, or from a clinical expert insight that mirrored a patient insight contained within the Patient Input Summary but was not used in the assessment.

## Discussion

This exploratory analysis shows that the use of patient insights can be traced through the course of the CDR process. Patient insights are used by CADTH reviewers to frame the assessment, and used by CDEC to interpret the assessment. Explicit references to patient insights within CDEC Recommendations tell only part of the story; implicit references to patient insights can be tracked, with access to the assessment reports, to better understand how patient perspectives are integrated into the CDR process.

HTA is about determining value. Clinicians, researchers, payers, and patients all have important, but potentially different, ideas on what should be considered, to determine value. Consider shopping for a new phone package. The “best deal” will depend upon what factors one values most: calling locally, calling nationally, frequency of Internet access, device included, device excluded, and/or cost. In the same way, the conclusion of a comparative assessment may depend upon what was considered. Patient groups may not use academic language, but if patient groups have the opportunity to contribute their insights prior to assessment protocol development, the assessment can be framed to reflect patient insights, in addition to those of clinical experts and researchers. If we are to make practice and policy decisions that reflect the values of patients, families, and citizens, we need to consider evidence from a variety of sources [4, 19].

If HTA is intended to address the indirect and unintended consequences of a technology, as well as its direct and intended effects [20], assessments should include the progress of recovery and sustainability of health. While the majority of patient insights in this study were shown to be integrated into the CDR process, CADTH could do better. Patient insights that fell within Porter’s first tier of survival and health status achieved (e.g., long life, symptom relief) were frequently included in all aspects of CDR drug assessments. The focus of the assessment is on immediate health status achieved with treatment. Porter’s second tier focuses on recovery, including treatment duration, side effects, and adherence. Within this second tier, although two-thirds of patient insights were included in the assessment protocol, only one-third had relevant trial data or was identified within the CDEC Recommendations document. The third-tier patient insights — independence, psychosocial quality of life, and avoiding further disease — were even less frequently addressed in protocols, in trials, or by CDEC. All address the long-term consequences of illness and its treatment.

Most drugs assessed by CDR are for chronic diseases. Patients living with chronic illnesses, such as chronic obstructive pulmonary disease (COPD), cystic fibrosis, Parkinson disease, and hypertension, will remain on the medication prescribed for many years, and perhaps for life. Treatment side effects such as fatigue, depression, nausea, or vein damage at the injection site, which might be considered minor when compared with survival, can loom large in a patient’s life, day after day [21]. The impact of treatment — taking time off work to receive an infusion, making repeated long journeys to hospital, going into debt to pay for medication — can have a substantial effect on both patients and their families. Patient insights included survival and relief of symptoms, but patient groups also identified what is important to be able to live a good life: not becoming a burden to one’s family, continuing to engage cognitively and emotionally, and avoiding further disease complications. These longer-term impacts of treatment offer valuable insights as clinical trial data are assessed for relevance within the realities of a Canadian health care setting.

We were surprised at the similarity of the insights that arose from the patient groups, given the wide range of drugs assessed. However, one can imagine that fear, frustration, or isolation might as easily be experienced by someone with rheumatoid arthritis as someone with multiple sclerosis or ulcerative colitis. If we had separated out symptoms by type (respiratory, cognitive, metabolic), we might have been able to explore differences between drug indications. Although symptom relief was the largest category, only 25 of the 119 insights focused on specific disease symptoms. Instead, we chose to explore differences between short-term and long-term patient insights, regardless of the type of illness.

### Implications for HTA

With a deliberately narrow recommendation framework, so the assessment can be completed within six months, CDR is not a classic HTA. Other HTA processes may have a more comprehensive deliberative framework, and supporting assessment reports, that explicitly seek to address ethical, legal, and social issues, in addition to clinical benefit and cost-effectiveness. However, as shown in this study, even an HTA with a narrow focus can incorporate patient insights.

CADTH reviewers consider and summarize, in their own words, the insights provided by patient groups in their completed templates. This summary, once patient groups have confirmed it contains all of their key insights, is shared with the external clinical experts and all team members. CADTH reviewers then translate patient groups’ descriptions of specific needs that are not met with current treatment, or their hopes regarding new therapies, into outcomes used within the relevant assessment protocols. For example, “According to the patient input received for this review, the control of COPD symptoms and prevention or minimization of the frequency and duration of exacerbations are key outcomes of importance to COPD patients.” [22]

Agencies that do not develop specific protocols, or a tailored scope, for each assessment may include one or more research questions built upon patient insights. Or explicit questions within a deliberative framework may prompt exploration of specific patient insights. Beyond using a written template to elicit patient insights, HTA agencies could directly involve individual patients in the assessment protocol development or in other stages of the assessment process to hear perspectives first-hand, and enable all participants in the assessment to directly ask questions to further understanding. In the absence of directly engaging patient groups, HTA agencies could include qualitative research articles describing the patient experience of living with a particular chronic condition, and/or their experience with a new drug or technology.

Another important use of patient insights is to prompt consideration of the real-world applicability of the clinical trial data. Recent research has demonstrated that patient perspectives add value to National Institute for Health and Care Excellence (NICE) Committee members in their interpretation of existing evidence — it enables them to consider the evidence “in a different light” [23].

Phase 3 clinical trials are typically designed around regional regulatory requirements, with one primary outcome and several secondary outcomes. The experimental drug is still often compared to placebo, rather than exploring differences between alternative therapies for the same indication, which might vary substantially from one health care setting to others. If patients are aware of and concerned about the side effects of currently available therapies, should not clinical trials capture and report on those known potential harms? As patients become more engaged in health research, through initiatives such as the James Lind Alliance, Core Outcome Measures in Effectiveness Trials (COMET), Patient and Community Engagement Research (PACER), and early scientific advice programs, the hope is that patient insights will be increasingly incorporated into clinical trials, thereby enabling more robust HTAs based on that trial data [24–27].

Understanding the specific ways in which these current therapies may fall short allows CADTH to better understand the value of the new drug, with respect to enabling patients to optimize their health and their lives, at the price proposed. Patients, in addition to clinical experts, can offer insights into different scenarios in which the new drug might be especially useful. However, in Canada, the decision to reimburse a drug happens around the time of regulatory approval, — not after patients and clinicians have used the drug for a year or two, as occurs in United Kingdom or Germany, for example. This means that few patients have any experience with the new drug or can speak to the real-world impact of improvements or side effects seen in trials.

Finally, descriptions of lived experiences can enliven the imagination of expert committee members and HTA researchers to spark deeper consideration of people and stories behind the numbers. As one former CDEC member described the value of patient input to CDR: “I’m very aware there are people on the other side of these submissions” [28].

Since June 2014, CADTH has added a qualitative methods specialist to staff, held additional training sessions for clinical reviewers and CDEC, and introduced feedback letters to patient groups. Each letter highlights what was most useful to CADTH reviewers and CDEC during the completed assessment and what would strengthen future patient group submissions. In 2016, CADTH is implementing a new CDEC recommendation framework, and a revised patient group input template. It is hoped that these activities will lead to a greater ability to better understand and integrate the perspectives and experiences provided by patient groups during drug assessments.

### Limitations

This analysis has a number of limitations. Patients’ perspectives are predominantly used in determining protocol outcomes, but they are also used by CADTH for context. Use of key informant interviews could strengthen the document analysis we conducted, especially to explore use of patients’ perspectives during the CDEC deliberations.

This exploratory analysis uses a mix of publicly available documents (CDEC Recommendations) and confidential reports available only to CADTH staff. Prior to April 2013, CDR reports were confidential. Since then, all assessment reports are published on CADTH’s website. The reports of 12 of the 30 drug assessments included in this study are, or will be, publicly available. As such, this exploratory analysis of CADTH’s process was conducted by CADTH. For recent drug assessments, the reports that include the assessment protocol, trial information, Patient Input Summary, and original patient input submissions are publicly available. This analysis could be repeated by other researchers, using more recent assessments.

Although 30 assessments were included, they reflect only what happened during that time frame, not necessarily use of patients’ perspectives overall, or since June 2014.

## Conclusions

This study has shown that patient insights can be incorporated as outcomes in HTA protocols and used in the interpretation of evidence. Patient insights are used by CADTH reviewers to frame the assessment, and used by CDEC in its interpretation of the evidence. Patients’ insights should not be “considered” in isolation, as they can inform many aspects of HTA such as assessment protocols, consideration of the real-world applicability of the clinical trial data and appreciation of a technology’s value for a specific scenario or subpopulation.

Porter’s Outcome Measures Hierarchy was used to quantify the observed juxtaposition between immediate outcomes of treatment seen in clinical trials and the experiences of, and insights raised by, patients living with a chronic condition. As HTA should address the indirect and unintended consequences of a technology, as well as its direct and intended effects, assessments should consider the progress of recovery and sustainability of health, in addition to survival and immediate health achieved. Patients can offer valuable insights on the longer-term impacts of treatment in the context of living with a chronic condition, ensuring that clinical trial data are interpreted for relevance within the realities of a Canadian health care setting.

## Abbreviations

CADTH, Canadian Agency for Drugs and Technologies in Health; CDR, CADTH Common Drug Review; CDEC, CADTH Canadian Drug Expert Committee; HTA, health technology assessment.

## Additional file

Additional file 1: Table S1. Inclusion of patient insights in 30 CADTH CDR assessments. (DOCX 15 kb)
