# Supplementary material for: Patients’ perspectives can be integrated in health technology assessments: an exploratory analysis of CADTH Common Drug Review
Source: Res Involv Engagem. 2016 Jun 7;2:21. doi: 10.1186/s40900-016-0036-9 (PMC5611639; doi:10.1186/s40900-016-0036-9)
Supplement: Additional file 1: Table S1. — Inclusion of patient insights in 30 CADTH CDR assessments. (DOCX 15 kb) [file 40900_2016_36_MOESM1_ESM.docx]

Patients’ perspectives can be integrated in health technology assessments: an exploratory analysis of CADTH Common Drug Review

| **Additional file 1: Table S1: Inclusion of patient insights in 30 CADTH CDR assessments** | | | | | |
| --- | --- | --- | --- | --- | --- |
| **CDR assessment** | **Patient insights** | **Included in CDR protocol** | **Included in drug trials** | **Included in**  **CDEC Recs** | **Highlighted as research gap** |
| Assessment | 4 | 3 | 2 | 2 | 0 |
| Assessment | 8 | 5 | 5 | 3 | 1 |
| Assessment | 0 | 0 | 0 | 0 | 0 |
| Assessment | 5 | 5 | 3 | 4 | 0 |
| Assessment | 5 | 3 | 2 | 3 | 0 |
| Assessment | 4 | 4 | 3 | 3 | 0 |
| Assessment | 4 | 4 | 4 | 4 | 1 |
| Assessment | 4 | 4 | 3 | 3 | 0 |
| Assessment | 5 | 2 | 1 | 3 | 0 |
| Assessment | 5 | 3 | 2 | 2 | 0 |
| Assessment | 0 | 0 | 0 | 0 | 0 |
| Assessment | 5 | 5 | 1 | 4 | 2 |
| Assessment | 6 | 4 | 3 | 4 | 0 |
| Assessment | 4 | 4 | 3 | 2 | 0 |
| Assessment | 3 | 1 | 1 | 1 | 0 |
| Assessment | 5 | 4 | 2 | 4 | 3 |
| Assessment | 0 | 0 | 0 | 0 | 0 |
| Assessment | 6 | 4 | 4 | 4 | 0 |
| Assessment | 6 | 4 | 2 | 2 | 0 |
| Assessment | 4 | 4 | 3 | 4 | 1 |
| Assessment | 4 | 3 | 3 | 2 | 1 |
| Assessment | 3 | 2 | 1 | 0 | 0 |
| Assessment | 4 | 3 | 1 | 3 | 0 |
| Assessment | 7 | 5 | 3 | 1 | 0 |
| Assessment | 4 | 2 | 2 | 2 | 0 |
| Assessment | 0 | 0 | 0 | 0 | 0 |
| Assessment | 4 | 4 | 3 | 4 | 0 |
| Assessment | 0 | 0 | 0 | 0 | 0 |
| Assessment | 5 | 4 | 3 | 2 | 0 |
| Assessment | 5 | 3 | 1 | 1 | 0 |
| **TOTAL** | **119** | **89** | **61** | **67** | **9** |
